# New Label-Free DNA Nanosensor Based on Top-Gated Metal–Ferroelectric–Metal Graphene Nanoribbon on Insulator Field-Effect Transistor: A Quantum Simulation Study

**DOI:** 10.3390/nano14242038

**Published:** 2024-12-19

**Authors:** Khalil Tamersit, Abdellah Kouzou, José Rodriguez, Mohamed Abdelrahem

**Affiliations:** 1 National School of Nanoscience and Nanotechnology, Abdelhafid Ihaddaden Scientific and Technological Hub, Sidi Abdellah, Algiers 16000, Algeria; 2 Laboratory of Inverse Problems, Modeling, Information and Systems (PIMIS), Université 8 Mai 1945 Guelma, Guelma 24000, Algeria; 3 Applied Automation and Industrial Diagnosis Laboratory (LAADI), Faculty of Science and Technology, Djelfa University, Djelfa 17000, Algeria; kouzouabdellah@ieee.org; 4 High-Power Converter Systems (HLU), Technical University of Munich (TUM), 80333 Munich, Germany; 5 Center for Energy Transition, Universidad San Sebastián, Santiago 8420524, Chile; jose.rodriguezp@uss.cl; 6 Electrical Engineering Department, Faculty of Engineering, Assiut University, Assiut 71516, Egypt

**Keywords:** deoxyribonucleic acid (DNA), field-effect transistor (FET), biosensors, quantum simulation, graphene nanoribbon (GNR), ferroelectric (FE), negative capacitance (NC), sensitivity

## Abstract

In this paper, a new label-free DNA nanosensor based on a top-gated (TG) metal–ferroelectric–metal (MFM) graphene nanoribbon field-effect transistor (TG-MFM GNRFET) is proposed through a simulation approach. The DNA sensing principle is founded on the dielectric modulation concept. The computational method employed to evaluate the proposed nanobiosensor relies on the coupled solutions of a rigorous quantum simulation with the Landau–Khalatnikov equation, considering ballistic transport conditions. The investigation analyzes the effects of DNA molecules on nanodevice behavior, encompassing potential distribution, ferroelectric-induced gate voltage amplification, transfer characteristics, subthreshold swing, and current ratio. It has been observed that the feature of ferroelectric-induced gate voltage amplification using the integrated MFM structure can significantly enhance the biosensor’s sensitivity to DNA molecules, whether in terms of threshold voltage shift or drain current variation. Additionally, we propose the current ratio as a sensing metric due to its ability to consider all DNA-induced modulations of electrical parameters, specifically the increase in on-state current and the decrease in off-state current and subthreshold swing. The obtained results indicate that the proposed negative-capacitance GNRFET-based DNA nanosensor could be considered an intriguing option for advanced point-of-care testing.

## 1. Introduction

Nanobiosensors based on field-effect transistors have garnered substantial interest due to their exceptional features, including label-free detection, miniaturization, compatibility with CMOS technology, and heightened sensitivity [1,2,3]. Notably, dielectric-modulated field-effect transistors (DMFETs) [4] have emerged as high-performance biosensors capable of detecting a diverse array of bio-measurands, ranging from avian influenza [5,6,7] to biotin–streptavidin binding [4], deoxyribonucleic acid (DNA) [8,9,10], human immunodeficiency virus (HIV) [11], and SARS-CoV-2 [12]. A key advantage of DMFET lies in its ability to detect both neutral and charged biomolecules [9], overcoming limitations observed in its ion-sensitive field-effect transistor (ISFET) counterpart [13]. Moreover, DMFETs exhibit scalability, versatility, and potential for improvement in terms of sensitivity, selectivity, and electrical performance [14,15,16]. Furthermore, DMFETs provide direct measurand detection while circumventing overlapping mechanisms, such as interactions between the measurand and channel that directly affect both electrostatics and transport [17]. Consequently, a multitude of experimental and computational studies have been undertaken to explore DMFETs’ potential, with a focus on optimizing performance [18,19,20], investigating new measurands [4,5,6,7,8,9,10,11,12], exploring innovative designs [21,22], proposing new computational approaches [23,24], and more. Furthermore, computational reports have highlighted the use of emerging 2D nanomaterials, such as Transition Metal Dichalcogenides (TMDs) [25], to enhance sensitivity and performance, thereby paving the way for the fabrication of innovative nanobiosensors. On the other hand, carbon-based materials, including graphene, graphene nanoribbons (GNRs), and carbon nanotubes, have found application as channels in DMFETs [10,26,27]. This choice was attributed to their heightened sensitivity to the electrostatic environment and their capacity to operate in the band-to-band tunneling regime [27,28,29] while providing ultra-high sensitivity in terms of drain current change. With the advent of the negative capacitance (NC) concept in FETs [30,31,32], several studies have put forth different DMFETs, leveraging a ferroelectric-based gating system to augment the biosensing capabilities of DMFETs [33,34,35,36,37,38,39]. However, as far as we are aware, there is no existing research that explores the performance outlook of a nanoscale FE n-i-n GNRFET as a label-free DNA sensor while proposing a new enhanced hybrid sensitivity.

In light of the recent advancements in DMFET technology, we introduce a novel label-free DNA nanosensor based on a top-gated metal–ferroelectric–metal graphene nanoribbon field-effect transistor (TG-MFM GNRFET). Our proposal is founded on a rigorous computational approach, combining quantum simulation [40,41,42] with the Landau–Khalatnikov theory [30]. This study comprehensively investigates the influence of DNA-induced dielectric changes on potential distribution, ferroelectric-induced gate voltage amplification, and transfer characteristics. The obtained results underscore the improved sensitivity and exceptional biosensing performance of the proposed label-free DNA sensor.

The remaining portion of this paper is structured as follows: Section 2 will outline the biosensing principle and DNA nanosensor structure. Section 3 will detail the computational approach employed in this work. Section 4 will present and discuss the results obtained. Finally, Section 5 will provide the concluding remarks for the paper.

## 2. Biosensor Structure and Biosensing Principle

**Figure 1:**
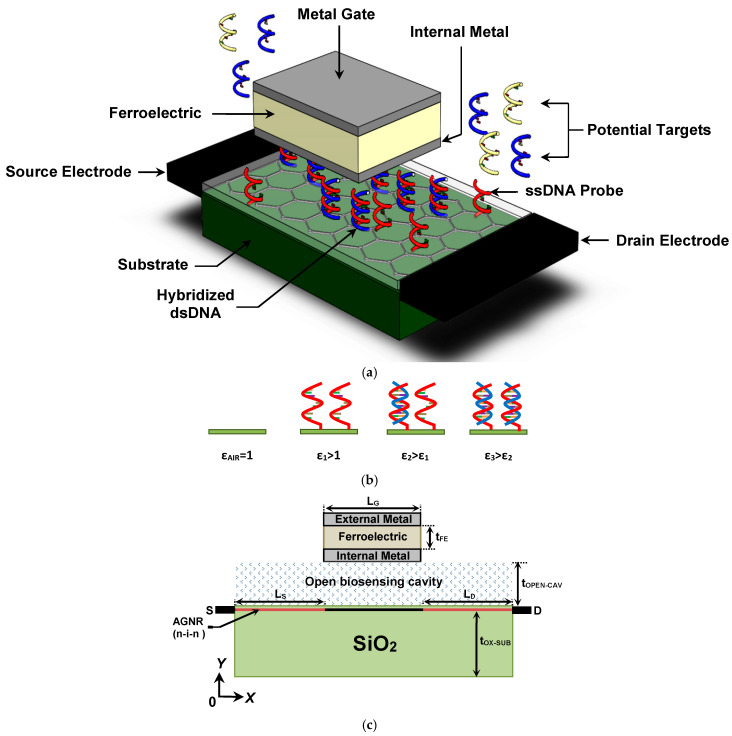
(**a**) Three-dimensional structure of the label-free DNA sensor based on TG-MFM GNRFET. (**b**) DNA detection based on the dielectric modulation concept. (**c**) Lengthwise cut view of the proposed nanoscale biosensor.

Figure 1a presents a three-dimensional (3D) perspective of the proposed TG-MFM GNRFET-based label-free DNA nanosensor. The nanosensor features an open cavity designed for DNA introduction and sensing. DNA detection relies on the DNA hybridization process, where single-stranded DNA (ssDNA) probes are initially introduced in the biosensing area and attached using self-assembled monolayer techniques [8,9,10]. These probes serve as selectors for specific DNA sequences through DNA hybridization. Utilizing the dielectric modulation concept [8,9,10], the introduction of ssDNA probes, the hybridization process, and the increase in hybridized DNA density are distinguishable through dielectric constant values, establishing a connection between biological and electrical mechanisms. It is worth noting that the range of the DNA-induced increment in the dielectric constant of the biosensing area is assumed to be 1–7, aligning with the experimentally observed range [9], while aiming to assess the proposed DNA nanosensor with low DNA concentrations reflecting small increments in the dielectric constant. In Figure 1a, non-hybridized ssDNA probes are shown to be attached to the thin insulator on the GNR channel, while others are shown to be hybridized. These neutral DNA-induced increments in dielectric constant, as shown in Figure 1b, induce electrostatic modulations in the FET, resulting in a shift in drain current (and its derivatives), which can be considered a metric [8,9,10]. Therefore, monitoring the FET drain current using appropriate readout circuits [7] allows for the extraction of relevant bio-information. It is noteworthy that our proposed FET-based biosensor features a compound gate made of an MFM design [43] to enhance the sensitivity of the DM FET-based DNA nanosensor through the ferroelectric-induced potential amplification concept [44,45,46,47,48]. Figure 1c illustrates a cross-sectional view of the proposed design, showcasing a top-gated armchair-edge GNR (AGNR) on an insulator FET with an open biosensing cavity and an MFM-based gate with hafnium zirconium oxide (HZO) ferroelectric. The substrate is made of SiO_2_, and the doping profile of the AGNR is typically considered n-i-n, with the intrinsic AGNR region located beneath the MFM gate. Note that n-i-n denotes a channel doping profile consisting of an n-type doped region, an intrinsic region, and another n-type doped region. The source (drain) contact is assumed to be ohmic. Parameters such as L_S(D)_, L_G_, t_OX-SUB_, t_OPEN-CAV_, and t_FE_ denote the length of the source (drain) reservoir, gate length, thickness of the insulator substrate, height of the open cavity, and ferroelectric thickness, respectively.

Conceptually, an equivalent circuit for the proposed sensor can be established considering the FE-based gate and the baseline nano-FET to be two spatially separated nano-components perfectly connected by a wire, simplifying the computational treatment [45,46,47]. Equivalently, the FE capacitance is connected in series with the baseline GNRFET.

## 3. Quantum Simulation Approach

**Figure 2:**
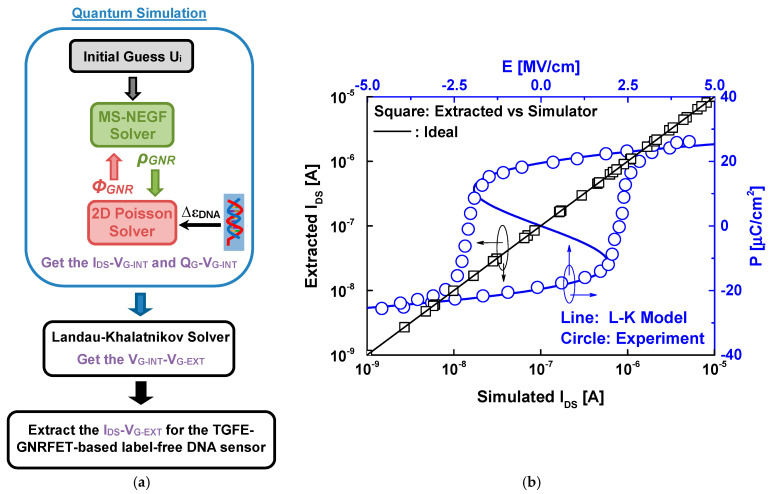
(**a**) Flowchart of the computational method used. (**b**) Drain current values from the literature and our simulator and the P–E proprieties from L–K theory and reported experiment data for the ferroelectric hafnium zirconium oxide.

Figure 2a illustrates the essential computational procedures required to simulate the TG-MFM GNRFET-based label DNA sensor. The flowchart comprises two primary computational blocks. The first block focuses on quantum mechanically simulating the baseline (without ferroelectric) top-gated GNRFET. This involves solving the Poisson equation, wherein DNA information is incorporated through the cavity dielectric constant, and employing the mode space NEGF self-consistently until convergence [49,50,51], as depicted in the quantum simulation block. The output of this convergence enables the extraction of drain current and gate charge as a function of the internal metal gate voltage.

The second block is dedicated to solving the Landau–Khalatnikov equation, utilizing the extracted gate charge to determine the voltage across the ferroelectric layer and allowing for the estimation of the external gate voltage [52,53]. Consequently, the drain current as a function of the external gate voltage becomes accessible [54,55], as illustrated in the last block of the computational flowchart in Figure 2a. It is worth noting that the simulations were carried out using a source code specifically developed in MATLAB 2023b software.

Figure 2b shows a comparison of the drain current from our simulator and some results reported in the literature [41,50,51] considering the same physical, electrical, and geometrical GNRFET parameters. As shown, we can clearly see the good agreement. Examining the same figure, we can observe the polarization–electric field characteristics derived from both the one-dimensional steady-state Landau–Khalatnikov equation and experimental results [48] for the ferroelectric HZO. The comparison reveals a close agreement, highlighting the accuracy, predictive capability, and generalizability of the Landau–Khalatnikov theory. Note that the experimentally calibrated Landau coefficients are taken to be α = −2.5 × 10^9^ Vm/C, β = 6 × 10^10^ Vm^5^/C^3^, and γ = 1.5 × 10^11^ Vm^9^/C^5^ [48]. In Appendix A, we provide the main equations used in the quantum simulation. For additional information and details concerning the NEGF simulation and the Landau–Khalatnikov modeling, we direct readers to some computational works [53,54,55,56,57,58].

## 4. Results and Discussion

**Figure 3:**
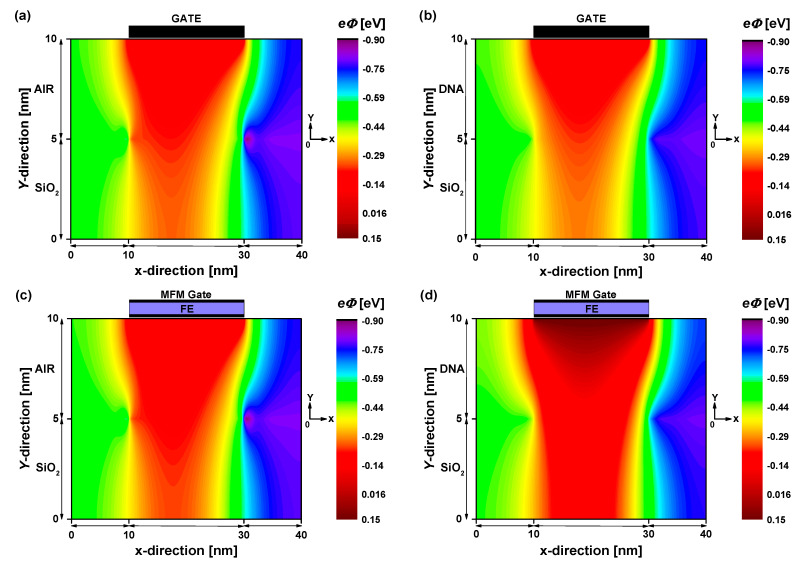
Two-dimensional electron potential distribution at V_DS_ = 0.3 V and V_GS_ = 0.1 V for baseline TG GNRFET-based biosensor (**top figures**) and TG-MFM GNRFET-based biosensor (**bottom figures).** (**a**,**c**) Empty open cavity. (**b**,**d**) Cavity filled with DNA molecules.

Figure 3 depicts the 2D potential distribution extracted from converged solutions of the NEGF–Poisson simulation for both the baseline and proposed biosensors under two sensing scenarios: the fresh biosensor (empty open cavity with ε_DNA_ = 1) and the active biosensor (cavity filled with DNA molecules with ε_DNA_ = 5). A low drain-to-source voltage, V_DS_ = 0.3 V, has been assumed to ensure low noise, low energy consumption, and low drain-induced barrier lowering. In the case of the baseline TG GNRFET-based DNA sensor (top figures), a subtle impact of DNA molecules on the electrostatic potential is observed, with slight modulations in drain current expected. Conversely, for the proposed DNA nanosensor (bottom figures), the influence of DNA molecules on the electrostatic potential is more pronounced, evident in an increased potential profile beneath the gate. Upon inspecting Figure 3a,c, illustrating the electrostatic potential of both designs with an empty open cavity, no remarkable change in the recorded electrostatic potential is noted despite the different methods of electrostatic gating (with and without FE). However, when the open sensing cavity is filled with DNA molecules (Figure 3b,d), the MFM device exhibits heightened sensitivity, in terms of electrostatic potential, to the DNA-induced increment in dielectric constant. This behavior suggests that FE-induced voltage amplification is significant when the MFM structure controls GNRFETs with high dielectric constant dielectrics, thus making ultra-high sensitivity achievable. It is worth noting that the FE-induced gate voltage amplification is maintained even with a large V_DS_, with some quantitative changes.

**Figure 4:**
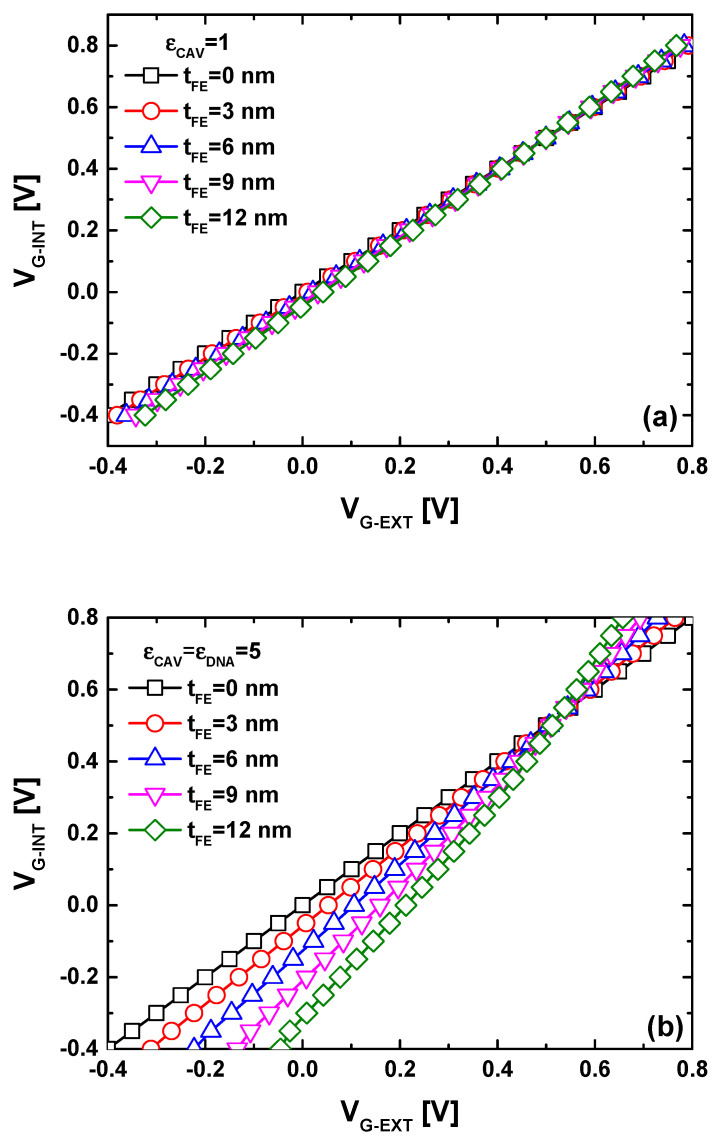
V_G-INT_ versus V_G-EXT_ for the proposed biosensor with different ferroelectric thicknesses considering (**a**) an empty and (**b**) filled sensing cavity.

Figure 4 presents a commonly used plot that illustrates the FE-induced gate voltage amplification by depicting the internal metal gate voltage as a function of the external gate voltage. In Figure 4a, it is evident that, in the case of an empty open biosensing cavity, a slight FE-induced gate voltage amplification is recorded, even with variations in ferroelectric thickness to enhance the FE-induced gate voltage amplification. In other words, there are no significant differences in terms of I_DS_-V_GS_ behavior between the baseline and MFM devices when the open biosensing cavity is empty (i.e., the reference condition). Figure 4b demonstrates that the FE-induced gate voltage amplification becomes significant when the open biosensing cavity is filled with DNA molecules, aligning with the electrostatic potential behaviors observed in Figure 3. Additionally, the plot indicates an increase in FE-induced gate voltage amplification with rising ferroelectric thickness. The results suggest that the detection of DNA molecules and relevant bio-events using the dielectric modulated GNRFET paradigm becomes more efficient with the MFM gating system, owing to the FE-induced gate voltage amplification that enhances biosensor sensitivity to the presence of DNA molecules. To quantitatively evaluate this significant finding, we subsequently assess the transfer characteristic and sensitivity of the proposed biosensor.

**Figure 5:**
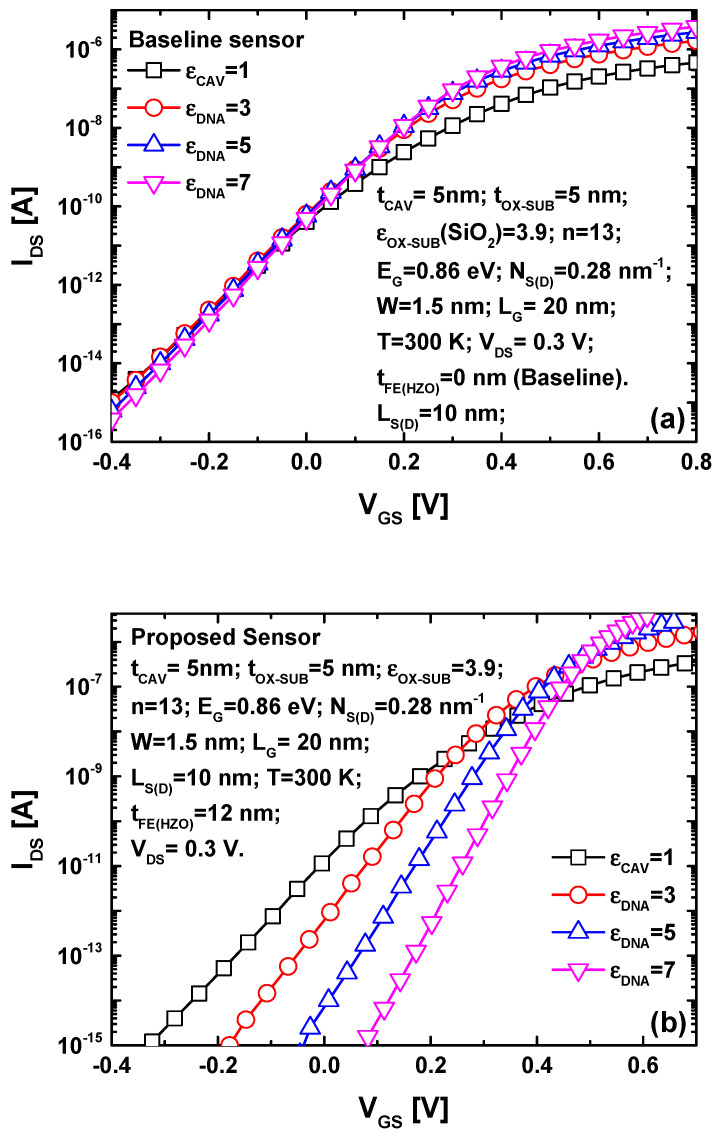
The I_DS_-V_GS_ characteristics for (**a**) the baseline and (**b**) the proposed biosensor considering different DNA dielectric constants.

Figure 5 shows the I_DS_-V_GS_ propriety for both nanosensors. The complete set of physical, dimensional, and electrical parameters employed in the simulations are indicated as inset in both figures. It is important to highlight that the indicated parameters are considered nominal, and we will explicitly emphasize any changes made to these parameters for the purpose of parametric analysis. Note that parameters (α, β, γ) represent the Landau parameters used in the voltage amplification assessment and were taken to be α = −2.5 × 10^9^ Vm/C, β = 6 × 10^10^ Vm^5^/C^3^, and γ = 1.5 × 10^11^ Vm^9^/C^5^. Figure 5a illustrates the impact of DNA-induced dielectric constant modulation on the I_DS_-V_GS_ transfer characteristic of the baseline GNRFET-based label-free DNA nanosensor. As depicted, the increase in the DNA dielectric constant within the sensing cavity slightly raises the on-state current and improves the subthreshold swing. However, there is no discernible change in the threshold voltage or subthreshold drain current that would classify them as sensing metrics. In the case of the proposed TG-MFM GNRFET-based biosensor, as shown in Figure 5b, significant alterations in the transfer characteristic are observed, whether in terms of the threshold voltage considering a fixed drain current [59] (e.g., the range between I_DS_ = 1 fA–1 pA) or subthreshold drain current considering a fixed gate voltage (e.g., V_GS_ = 0.1 V). The recorded increase in drain current sensitivity is attributed to the FE-induced gate voltage amplification, which is notable in the presence of DNA (i.e., ε_CAV_ > 1) and very slight in the case of an empty sensing cavity. Examining Figure 5b, we can also observe that the on-state current increases (and the off-state current decreases) with an increasing DNA dielectric constant, making the subthreshold current slope steeper.

**Figure 6:**
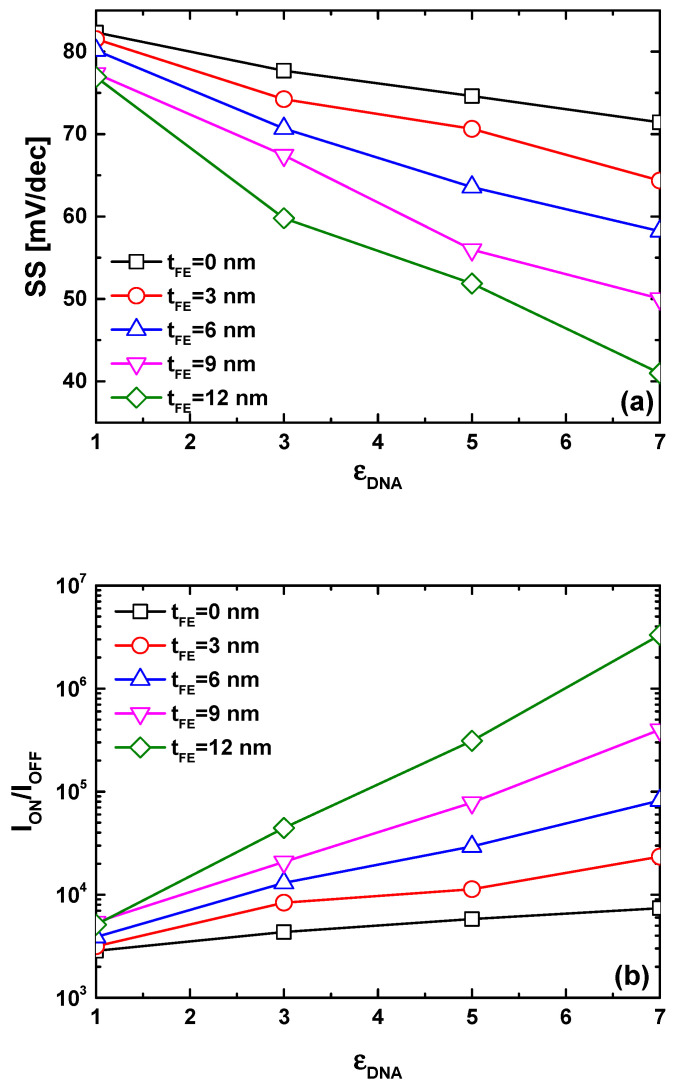
Subthreshold swing (**a**) and current ratio (**b**) as functions of DNA dielectric constant for the TG-MFM GNRFET-based DNA sensor.

Figure 6a illustrates the behavior of subthreshold swing versus DNA dielectric constant for the proposed biosensor considering different ferroelectric thicknesses. By definition, the subthreshold swing can be seen as the required gate voltage to change the drain current by about one order of magnitude [60,61,62]. It is evident that the subthreshold swing decreases with an increase in the DNA dielectric constant, as observed in Figure 5b. Notably, an increase in ferroelectric thickness enables the attainment of steeper subthreshold swing values, making subthermionic subthreshold swing achievable. This outcome is anticipated due to the FE-induced gate voltage amplification, which accelerates the device switching [63,64,65]. To comprehensively capture the collective effects of DNA dielectric constant increment on transfer characteristics (i.e., I_ON_ increasing, I_OFF_ decreasing, SS lowering), we employ the I_ON_/I_OFF_ current ratio as a sensing metric while considering the power supply voltage (V_DD_) equal to the drain-to-source voltage [66,67,68]. Our choice of this metric is grounded in its sensitivity to changes in on-current, off-current, and subthreshold swing. In our case, all recorded trends (i.e., I_ON_ increasing, I_OFF_ decreasing, SS lowering) contribute to an increase in the current ratio, rendering it an innovative sensing metric. Figure 6b demonstrates that the current ratio increases with an increase in DNA dielectric constant, aligning with the recorded trends of I_ON_ increasing, I_OFF_ decreasing, and SS decreasing under ε_DNA_ increment. It is noteworthy that biosensors with a thicker ferroelectric layer exhibit higher sensitivity compared to those endowed with thin FE material, thereby rendering DNA events (e.g., ssDNA density, DNA hybridization, dsDNA concentration, etc.) more distinguishable, as clearly shown in the same figure.

As a potential direction for further investigation, bio-inspired optimizers could be applied in conjunction with the quantum simulation approach and the Landau–Khalatnikov theory to identify the optimal parameters—including MFM-based gate design, FET transducer configuration, biosensing cavity, and DNA sizes—that enhance biosensing performance [69,70,71]. In this context, the optimization phase could also explore the junctionless paradigm, different ferroelectric materials, various channel nanomaterials, and diverse gate geometries [72,73].

## 5. Conclusions

In this paper, we successfully proposed ferroelectric-induced gate voltage amplification to enhance the performance and sensitivity of a top-gated GNRFET-based label-free DNA nanosensor, employing a rigorous computational approach. This approach integrates quantum mechanical simulation with the Landau–Khalatnikov equation. The dielectric modulation concept, involving DNA-induced dielectric increment, is intricately embedded in the Poisson solver, meticulously considering the relevant nodes in the open biosensing area. Our proposed nanosensor features a compound gate based on a metal–ferroelectric–metal structure, aiming to magnify the effects of DNA-induced dielectric constant increment on the electrostatics and transport of the nanobiosensor. The simulation results unequivocally demonstrate a significant improvement in both electrical and sensing performance. Furthermore, by accounting for the impact of DNA-induced dielectric increment on the device’s figure of merits (i.e., I_ON_, I_OFF_, SS), we introduce the I_ON_/I_OFF_ current ratio as a biosensing metric. This metric is chosen because the DNA-induced dielectric increment boosts this ratio by reducing the subthreshold swing, decreasing the leakage current, and increasing the on-state current, thus implicitly consolidating three sensing metrics into one comprehensive measure. The obtained results explicitly showcase the high performance of our proposed sensor, encompassing label-free DNA sensing, CMOS compatibility, compact size, low-energy consumption, and improved sensitivity.

## Data Availability

The data that support the findings of this study are available from the first corresponding author (K.T.) upon reasonable request.

## Appendix A

In this appendix, we detail the key equations employed in the self-consistent NEGF–Poisson coupling integrated with the Landau–Khalatnikov theory. As described in Section 3, the initial step involves the quantum simulation of the baseline GNRFET-based biosensor (i.e., without the MFM structure). This process necessitates the computation of the retarded Green’s function, *G*, which can be represented as [26]

(A1) $$G(E)={\left[(E+i\eta^{+})I-H-\Sigma_{S}-\Sigma_{D}\right]}^{-1}$$

where *E* represents the energy, *I* denotes the identity matrix, and *η*^+^ is an infinitesimal positive value. The Hamiltonian *H* is derived using the atomistic nearest-neighbor tight-binding approximation. In the MS representation, the source (drain) self-energy Σ*_S_*_(*D*)_ for the *q*th mode can be calculated as follows [49]

(A2) $$\Sigma_{Sq\;(Dq)}=\frac{\alpha_{1(M)}+\sqrt{{\left[\alpha_{1(M)}\right]}^{2}-4{\left(E-U_{1(M)}\right)}^{2}b_{1q}^{2}}}{2(E-U_{1(M)})}$$

with

(A3) $$\alpha_{1(M)}={\left(E-U_{1(M)}\right)}^{2}+b_{1q}^{2}-b_{2q}^{2}$$

where *t*_0_ = 2.7 eV is the nearest neighbor tight-binding parameter and Δ = 0.12 is a fitting parameter accounting for edge bond relaxation. The hopping parameters between the carbon lines are defined as *b*_1*q*_ = *t*_0_ + 4Δ*t*_0_*sin*^2^[*qπ*/(*n* + 1)]/(*n* + 1) and *b*_2*q*_ = 2*t*_0_*cos*(*πq*/(*n* + 1)) [49]*. U*_1(*M*)_ represents the electrostatic potential at the first (last) GNR lattice column, which is assumed to be connected to the source (drain) contact. The local density of states *D_S_*_(*D*)_ can now be determined using the following expression:

(A4) $$D_{S(D)}=G\Gamma_{S(D)}G^{+}$$

where $\Gamma_{S(D)}=i(\Sigma_{S(D)}-\Sigma_{S(D)}^{+})$ represents the energy level broadening caused by the source (drain) contact. Calculating the aforementioned NEGF quantities enables the estimation of the charge density in the armchair-edge GNR channel through the following equation [49]:

(A5) $$\begin{array}{l} Ne=\int_{-\infty }^{+\infty }dE\;\mathrm{sgn}\left[E-E_{N}\right]\left\{D_{S}(E)\right.f(\mathrm{sgn}\left[E-E_{N}\right](E-E_{FS}))\\ \;\;\;\;\;\;\;\;\;\;+D_{D}(E)f(\mathrm{sgn}\left[E-E_{N}\right](E-E_{FD})\left.)\right\} \end{array}$$

Here, *sgn* denotes the sign function, and *f*(*sgn*[*E* − *E_N_*].(*E* − *E_FS_*_(*D*)_)) is the source (drain) Fermi function corresponding to the Fermi level *E_FS_*_(*D*)_, with *E_N_* being the charge neutrality level [49]. In the NEGF simulation approach, computing the charge density in the self-consistent NEGF–Poisson coupling requires an approximation of the electrostatics, which can be estimated by solving the Poisson equation expressed as [10]

(A6) $$\nabla^{2}U=\frac{-q}{\varepsilon }\rho$$

where *U* denotes the electrostatic potential, *ε* represents the dielectric constant, and *ρ* describes the distribution of net charge density. Considering the finite difference method (FDM) and the dielectric modulation concept, the dielectric constant *ε* is assigned as follows: *ε_AIR_* = 1 in the region filled with air, *ε_DNA_* > 1 in the region occupied by DNA molecules, and *ε_OX_* in the oxide region [10,26,27]. In the Poisson solver based on the FDM, the Neumann boundary condition is applied to all external interfaces, including the source and drain, except at the gate metal level, where the Dirichlet boundary condition is used. After achieving self-consistency, the channel current can be calculated using the following formula [49]:

(A7) $$I=\frac{2q}{h}\int dE\;T(E)\left[f(E-E_{FS})-f(E-E_{FD})\right]$$

In this expression, *q* signifies the electron charge, *h* stands for Planck’s constant, *T*(*E*) = *Tr*(*Γ_S_GΓ_D_G*^+^) is the transmission coefficient, and *Tr* indicates the trace operator. For the numerical modeling of the FE FETs, it is necessary to use the Landau–Khalatnikov equation given by [30]

(A8) $$\rho \frac{dP}{dt}+\nabla_{P}U=0$$

where *ρ* indicates the resistivity, *P* represents the ferroelectric polarization, *t* is time, and *U* denotes the free energy of the ferroelectric system, which can be expressed as follows:

(A9) $$U=\alpha P^{2}+\beta P^{4}+\gamma P^{6}-EP$$

where (*α*, *β*, *γ*) are the parameters of the ferroelectric material and *E* is the electric field externally applied to the ferroelectric layer. Using Equations (A8) and (A9), we obtain

(A10) $$E=2\alpha P+4\beta P^{3}+6\gamma P^{5}+\rho \frac{dP}{dt}$$

By taking *Q* = *P* and *V_FE_* = *Et_FE_* while considering the FE steady-state polarization (i.e., *dP*/*dt* = 0), we obtain the *Q*-*V* equation [30,47,53]

*V_FE_ =* 2*αt_FE_Q_G_ +* 4*βt_FE_Q_G_*^3^
*+* 6*γt_FE_Q_G_*^5^ (A11)

where *Q_G_* denotes the gate charge of the baseline dielectric modulated GNRFET-based biosensor and *V_FE_* represents the voltage across the ferroelectric. The external gate voltage applied to the metal–ferroelectric–metal structure, *V_GS_*, can be determined as [52]

*V_GS_ = V_INT_ + V_FE_* (A12)

where *V_INT_* represents the voltage of internal metal, which corresponds to the gate voltage of the baseline GNRFET-based DNA sensor.

## References

[1] Chen K.I., Li B.R., Chen Y.T. (2011). Silicon nanowire field-effect transistor-based biosensors for biomedical diagnosis and cellular recording investigation. Nano Today.

[2] Hao R., Liu L., Yuan J., Wu L., Lei S. (2023). Recent Advances in Field Effect Transistor Biosensors: Designing Strategies and Applications for Sensitive Assay. Biosensors.

[3] Schöning M.J., Poghossian A. (2002). Recent advances in biologically sensitive field-effect transistors (BioFETs). Analyst.

[4] Im H., Huang X.-J., Gu B., Choi Y.-K. (2007). A dielectric-modulated field-effect transistor for biosensing. Nat. Nanotechnol..

[5] Gu B., Park T.J., Ahn J., Huang X., Lee S.Y., Choi Y. (2009). Nanogap Field-Effect Transistor Biosensors for Electrical Detection of Avian Influenza. Small.

[6] Lee K.-W., Choi S.-J., Ahn J.-H., Moon D.-I., Park T.J., Lee S.Y., Choi Y.-K. (2010). An underlap field-effect transistor for electrical detection of influenza. Appl. Phys. Lett..

[7] Im M., Ahn J.-H., Han J.-W., Park T.J., Lee S.Y., Choi Y.-K. (2010). Development of a point-of-care testing platform with a nanogap-embedded separated double-gate field effect transistor array and its readout system for detection of avian influenza. IEEE Sensors J..

[8] Kim C.-H., Jung C., Lee K.-B., Park H.G., Choi Y.-K. (2011). Label-free DNA detection with a nanogap embedded complementary metal oxide semiconductor. Nanotechnology.

[9] Kim C.-H., Jung C., Park H.G., Choi Y.-K. (2008). Novel dielectric-modulated field-effect transistor for label-free DNA detection. Biochip J..

[10] Tamersit K., Djeffal F. (2016). Double-Gate Graphene Nanoribbon Field-Effect Transistor for DNA and Gas Sensing Applications: Simulation Study and Sensitivity Analysis. IEEE Sensors J..

[11] Kim J.Y., Ahn J.H., Moon D.I., Park T.J., Lee S.Y., Choi Y.K. (2014). Multiplex electrical detection of avian influenza and human immunodeficiency virus with an under-lap-embedded silicon nanowire field-effect transistor. Biosens. Bioelectron..

[12] Yadav S., Gedam A., Tirkey S. (2021). A dielectric modulated biosensor for SARS-CoV-2. IEEE Sens. J..

[13] Bergveld P. (2003). Thirty years of ISFETOLOGY. Sens. Actuators B Chem..

[14] Narang R., Reddy K.V.S., Saxena M., Gupta R.S., Gupta M. (2012). A Dielectric-modulated tunnel-FET-based biosensor for label-free detection: Analytical modeling study and sensitivity analysis. IEEE Trans. Electron Devices.

[15] Narang R., Saxena M., Gupta M. (2015). Comparative Analysis of Dielectric-Modulated FET and TFET-Based Biosensor. IEEE Trans. Nanotechnol..

[16] Choi J.-M., Han J.-W., Choi S.-J., Choi Y.-K. (2010). Analytical Modeling of a Nanogap-Embedded FET for Application as a Biosensor. IEEE Trans. Electron Devices.

[17] Chen X., Guo Z., Yang G.-M., Li J., Li M.-Q., Liu J.-H., Huang X.-J. (2010). Electrical nanogap devices for biosensing. Mater. Today.

[18] Ghosh S., Chattopadhyay A., Tewari S. (2020). Optimization of Hetero-Gate-Dielectric Tunnel FET for Label-Free Detection and Identification of Biomolecules. IEEE Trans. Electron Devices.

[19] Sehgal H.D., Pratap Y.E., Gupta M., Kabra S. (2021). Performance Analysis and Optimization of Under-Gate Dielectric Modulated Junctionless FinFET Biosensor. IEEE Sensors J..

[20] Kim S., Ahn J.-H., Park T.J., Lee S.Y., Choi Y.-K. (2010). Comprehensive study of a detection mechanism and optimization strategies to improve sensitivity in a nanogap-embedded biotransistor. J. Appl. Phys..

[21] Anvarifard M.K., Ramezani Z., Amiri I.S., Tamersit K., Nejad A.M. (2020). Profound analysis on sensing performance of Nanogap SiGe source DM-TFET biosensor. J. Mater. Sci. Mater. Electron..

[22] Kannan N., Kumar M.J. (2013). Dielectric-Modulated Impact-Ionization MOS Transistor as a Label-Free Biosensor. IEEE Electron Device Lett..

[23] Ajay, Narang R., Saxena M., Gupta M. (2017). Modeling and Simulation Investigation of Sensitivity of Symmetric Split Gate Junctionless FET for Biosensing Application. IEEE Sensors J..

[24] Ajay, Narang R., Saxena M., Gupta M. (2015). Drain Current Model of a Four-Gate Dielectric Modulated MOSFET for Application as a Biosensor. IEEE Trans. Electron Devices.

[25] Rahman E., Shadman A., Ahmed I., Khan S.U.Z., Khosru Q.D.M. (2018). A physically based compact I–V model for monolayer TMDC channel MOSFET and DMFET biosensor. Nanotechnology.

[26] Tamersit K., Djeffal F. (2019). Carbon Nanotube Field-Effect Transistor with Vacuum Gate Dielectric for Label-Free Detection of DNA Molecules: A Computational Investigation. IEEE Sensors J..

[27] Tamersit K. (2024). Dielectric-modulated junctionless carbon nanotube field-effect transistor as a label-free DNA nanosensor: Achieving ultrahigh sensitivity in the band-to-band tunneling regime. IEEE Sensors J..

[28] Avouris P., Chen Z., Perebeinos V. (2007). Carbon-based electronics. Nat. Nanotechnol..

[29] Tamersit K., Ramezani Z., Amiri I.S. (2022). Improved performance of sub-10-nm band-to-band tunneling n-i-n graphene nanoribbon field-effect tran-sistors using underlap engineering: A quantum simulation study. J. Phys. Chem. Solids.

[30] Salahuddin S., Datta S. (2007). Use of Negative Capacitance to Provide Voltage Amplification for Low Power Nanoscale Devices. Nano Lett..

[31] Si M., Su C.-J., Jiang C., Conrad N.J., Zhou H., Maize K.D., Qiu G., Wu C.-T., Shakouri A., Alam M.A. (2017). Steep-slope hysteresis-free negative capacitance MoS_2_ transistors. Nat. Nanotechnol..

[32] Jooq M.K.Q., Moaiyeri M.H., Tamersit K. (2022). A New Design Paradigm for Auto-Nonvolatile Ternary SRAMs Using Ferroelectric CNTFETs: From Device to Array Architecture. IEEE Trans. Electron Devices.

[33] Singh S., Agnihotri S.K., Bagga N., Samajdar D.P. (2024). Assessment of the Biosensing Capabilities of SiGe Heterojunction Negative Capacitance-Vertical Tunnel Field-Effect Transistor. ACS Appl. Bio Mater..

[34] Tamersit K., Bourouba H. Negative capacitance junctionless graphene nanoribbon tunneling FET as DNA nanosensor: A quantum simulation-based proposal. Proceedings of the 2024 IEEE Workshop on Complexity in Engineering (COMPENG).

[35] Vanlalawmpuia K., Ghosh P. (2023). Performance assessment of dielectrically modulated negative capacitance germanium source vertical tunnel FET biosensor for detection of breast cancer cell lines. AEU Int. J. Electron. Commun..

[36] Dixit A., Samajdar D.P., Chauhan V. (2021). Sensitivity Analysis of a Novel Negative Capacitance FinFET for Label-Free Bio-sensing. IEEE Trans. Electron Devices.

[37] Das B., Bhowmick B. (2023). Dielectrically modulated ferroelectric-TFET (Ferro-TFET) based biosensors. Mater. Sci. Eng. B.

[38] Ghosh P., Bhowmick B. (2022). Performance enhancement of a FET device with ferroelectric tunnel junction and its application as a biosensor. J. Comput. Electron..

[39] Pathak Y., Malhotra B.D., Chaujar R. (2022). Detection of biomolecules in dielectric modulated double metal below ferroelectric layer FET with improved sensitivity. J. Mater. Sci. Mater. Electron..

[40] Tamersit K. (2020). Performance enhancement of an ultra-scaled double-gate graphene nanoribbon tunnel field-effect transistor using channel doping engineering: Quantum simulation study. AEU Int. J. Electron. Commun..

[41] Yousefi R., Shabani M., Arjmandi M., Ghoreishi S. (2013). A computational study on electrical characteristics of a novel band-to-band tunneling graphene nanoribbon FET. Superlattices Microstruct..

[42] Tamersit K. (2019). A computational study of short-channel effects in double-gate junctionless graphene nanoribbon field-effect transistors. J. Comput. Electron..

[43] Tu L., Wang X., Wang J., Meng X., Chu J. (2018). Ferroelectric Negative Capacitance Field Effect Transistor. Adv. Electron. Mater..

[44] Alam M.A., Si M., Ye P.D. (2019). A critical review of recent progress on negative capacitance field-effect transistors. Appl. Phys. Lett..

[45] Behbahani F., Jooq M.K.Q., Moaiyeri M.H., Tamersit K. (2021). Leveraging Negative Capacitance CNTFETs for Image Processing: An Ultra-Efficient Ternary Image Edge Detection Hardware. IEEE Trans. Circuits Syst. I Regul. Pap..

[46] Tamersit K. (2021). Improved switching performance of nanoscale p-i-n carbon nanotube tunneling field-effect transistors using met-al-ferroelectric-metal gating approach. ECS J. Solid State Sci. Technol..

[47] You W.-X., Tsai C.-P., Su P. (2018). Short-Channel Effects in 2D Negative-Capacitance Field-Effect Transistors. IEEE Trans. Electron Devices.

[48] Saha A.K., Gupta S.K. (2020). Multi-Domain Negative Capacitance Effects in Metal-Ferroelectric-Insulator-Semiconductor/Metal Stacks: A Phase-field Simulation Based Study. Sci. Rep..

[49] Zhao P., Guo J. (2009). Modeling edge effects in graphene nanoribbon field-effect transistors with real and mode space methods. J. Appl. Phys..

[50] Zhao P., Chauhan J., Guo J. (2009). Computational study of tunneling transistor based on graphene nanoribbon. Nano Lett..

[51] Yoon Y., Fiori G., Hong S., Iannaccone G., Guo J. (2008). Performance comparison of graphene nanoribbon FETs with schottky contacts and doped reservoirs. IEEE Trans. Electron Devices.

[52] Seo J., Lee J., Shin M. (2017). Analysis of Drain-Induced Barrier Rising in Short-Channel Negative-Capacitance FETs and Its Ap-plications. IEEE Trans. Electron Devices.

[53] Tamersit K., Moaiyeri M.H., Jooq M.K.Q. (2022). Leveraging negative capacitance ferroelectric materials for performance boosting of sub-10 nm graphene nanoribbon field-effect transistors: A quantum simulation study. Nanotechnology.

[54] Tamersit K., Jooq M.K.Q., Moaiyeri M.H. (2021). Analog/RF performance assessment of ferroelectric junctionless carbon nanotube FETs: A quantum simulation study. Phys. E Low-Dimens. Syst. Nanostruct..

[55] Tamersit K., Kouzou A., Bourouba H., Kennel R., Abdelrahem M. (2022). Synergy of electrostatic and chemical doping to improve the performance of junctionless carbon nanotube tunneling field-effect transistors: Ultrascaling, energy-efficiency, and high switching performance. Nanomaterials.

[56] Khorshidsavar A., Ghoreishi S.S., Yousefi R. (2018). A Computational Study of an Optimized MOS-Like Graphene Nano Ribbon Field Effect Transistor (GNRFET). ECS J. Solid State Sci. Technol..

[57] Ghoreishi S.S., Yousefi R. (2017). A computational study of a novel graphene nanoribbon field effect transistor. Int. J. Mod. Phys. B.

[58] Datta S. (2005). Quantum Transport: Atom to Transistor.

[59] Ortiz-Conde A., Garcia-Sanchez F., Liou J.J., Cerdeira A., Estrada M., Yue Y. (2002). A review of recent MOSFET threshold voltage extraction methods. Microelectron. Reliab..

[60] Tamersit K. (2021). New nanoscale band-to-band tunneling junctionless GNRFETs: Potential high-performance devices for the ul-trascaled regime. J. Comput. Electron..

[61] Choi W., Jin B., Shin S., Do J., Son J., Kim K., Lee J.-S. (2023). Highly Sensitive Detection of Urea Using Si Electrolyte-Gated Transistor with Low Power Consumption. Biosensors.

[62] Kim D., Jin B., Kim S.-A., Choi W., Shin S., Park J., Shim W.-B., Kim K., Lee J.-S. (2022). An Ultrasensitive Silicon-Based Electrolyte-Gated Transistor for the Detection of Peanut Allergens. Biosensors.

[63] Zhirnov V.V., Cavin R.K. (2008). Negative capacitance to the rescue?. Nat. Nanotechnol..

[64] Jiao H., Wang X., Wu S., Chen Y., Chu J., Wang J. (2023). Ferroelectric field effect transistors for electronics and optoelectronics. Appl. Phys. Rev..

[65] Khan A.I., Keshavarzi A., Datta S. (2020). The future of ferroelectric field-effect transistor technology. Nat. Electron..

[66] Yao X., Xie R., Zan X., Su Y., Xu P., Liu W. (2023). A Novel Image Encryption Scheme for DNA Storage Systems Based on DNA Hybridization and Gene Mutation. Interdiscip. Sci. Comput. Life Sci..

[67] Chu L., Su Y., Zan X., Lin W., Yao X., Xu P., Liu W. (2024). A Deniable Encryption Method for Modulation-Based DNA Storage. Interdiscip. Sci. Comput. Life Sci..

[68] Hou X., Xin L., Fu Y., Na Z., Gao G., Liu Y., Xu Q., Zhao P., Yan G., Su Y. (2023). A self-powered biomimetic mouse whisker sensor (BMWS) aiming at terrestrial and space objects perception. Nano Energy.

[69] Jakšić Z., Devi S., Jakšić O., Guha K. (2023). A Comprehensive Review of Bio-Inspired Optimization Algorithms Including Applications in Microelectronics and Nanophotonics. Biomimetics.

[70] Trojovský P., Dehghani M. (2023). A new bio-inspired metaheuristic algorithm for solving optimization problems based on walruses behavior. Sci. Rep..

[71] Zhang D., Han C., Zhang H., Zeng B., Zheng Y., Shen J., Wu Q., Zeng G. (2021). The Simulation Design of Microwave Absorption Performance for the Multi-Layered Carbon-Based Nano-composites Using Intelligent Optimization Algorithm. Nanomaterials.

[72] Nowbahari A., Roy A., Marchetti L. (2020). Junctionless Transistors: State-of-the-Art. Electronics.

[73] Wang M. (2024). A Review of Reliability in Gate-All-Around Nanosheet Devices. Micromachines.

